# Interaction of macro- and microvascular function underlies brachial artery flow-mediated dilation in humans

**DOI:** 10.1152/ajpheart.00158.2024

**Published:** 2024-05-24

**Authors:** Clare E. Thorn, Phillip E. Gates, Francesco Casanova, Alessandro Ramalli, Piero Tortoli, Carlo Palombo, Angela C. Shore, Kunihiko Aizawa

**Affiliations:** ^1^Department of Clinical and Biomedical Sciences, University of Exeter Medical School, Exeter, United Kingdom; ^2^National Institute of Health and Care Research Exeter Clinical Research Facility, Exeter, United Kingdom; ^3^Department of Health and Care Professions, Faculty of Health and Life Sciences, University of Exeter, Exeter, United Kingdom; ^4^Department of Information Engineering, University of Florence, Florence, Italy; ^5^Department of Surgical, Medical, Molecular Pathology and Critical Care Medicine, University of Pisa, Pisa, Italy

**Keywords:** aging, blood flow, cardiovascular, endothelium, oxygenation

## Abstract

Brachial artery flow-mediated dilation (BAFMD) is induced by hyperemic wall shear rate (WSR) following forearm ischemia. In older adults, there appears to be a reduced brachial hyperemic WSR and altered stimulus-response relationship compared with young adults. However, it is unclear if an altered forearm microvascular response to ischemia influences brachial hyperemic WSR in older adults. We determined associations between brachial hyperemic WSR and forearm skeletal muscle oxygen saturation in young and older adults. Healthy young (*n* = 17, 29 ± 7 yr) and older (*n* = 32, 65 ± 4 yr) adults participated in the study. BAFMD by a multigate spectral Doppler system and forearm skeletal muscle oxygen saturation by near-infrared spectroscopy were concurrently measured. When compared with the young, older adults showed reduced oxygen extraction kinetics (OE, 0.15 [0.12–0.17] vs. 0.09 [0.05–0.12]%s^−1^) and magnitude (So_2deficit_, 3,810 ± 1,420 vs. 2,723 ± 1,240%s) during ischemia, as well as oxygen resaturation kinetics (So_2slope_, 2.5 ± 0.7 vs. 1.7 ± 0.7%s^−1^) upon reperfusion (all *P* < 0.05). When OE in the young and So_2slope_ in older adults were stratified by their median values, young adults with OE above the median had greater hyperemic WSR parameters compared with those below the median (*P* < 0.05), but So_2slope_ in older adults did not show clear differences in hyperemic WSR parameters between those above/below the median. This study demonstrates that, in addition to a reduced microvascular response to ischemia, there may be a dissociation between microvascular response to ischemia and brachial hyperemic WSR in older adults, which may result in a further impairment of BAFMD in this cohort.

**NEW & NOTEWORTHY** Microvascular response to ischemia and subsequent reperfusion is diminished in older adults compared with the young. Furthermore, there appears to be a dissociation between the microvascular response to ischemia and brachial hyperemic WSR in older adults, which may further disturb the BAFMD process in this cohort. A reduced BAFMD in older adults may be a result of multiple alterations occurring both at macro- and microcirculation.

## INTRODUCTION

Brachial artery flow-mediated dilation (BAFMD) has been an essential tool for cardiovascular research and is used to elucidate the role that the vascular endothelium plays in health and disease (1). BAFMD requires a cascade of events where each event becomes a stimulus for the next one. Hyperemic wall shear rate (WSR) induced by a sudden release of the forearm cuff following a 5-min arterial occlusion is the stimulus for brachial artery (BA) vasodilation. However, forearm microvasculature also responds to this temporal ischemic stimulus (2) and the response is likely to affect hyperemic WSR. An oxygen supply-demand mismatch results in microvascular vasodilation during ischemia, and the extent of vasodilation and reperfusion following cuff release influences the magnitude of BA WSR (3). It is thus plausible that an altered microvascular response to ischemia and/or altered microvascular reperfusion response after cuff release may influence BA hyperemic WSR. However, evidence to support this contention remains unclear. This is critical because the identification of microvascular parameters that influence BA hyperemic WSR will enhance our understanding of the entire stimulus/response relationship of BAFMD and could help delineate the origin of diminished hyperemic WSR typically observed in healthy older adults (4). Furthermore, a number of reports have shown the association between BAFMD (or BA reactive hyperemia) and microvascular parameters that are related to oxygen extraction during ischemia and oxygen resaturation during reperfusion (5–7). The established technique of near-infrared spectroscopy (NIRS) noninvasively assesses the oxygenation status and hemodynamics of the microcirculation and provides a measure of oxygen supply/demand in the tissue. However, it is currently unknown if those microvascular parameters are associated with BA hyperemic WSR parameters.

Accordingly, we determined NIRS-derived oxygen saturation of the forearm skeletal muscle concurrently with a standard BAFMD procedure. We first determined associations between microcirculatory oxygen saturation parameters and BA hyperemic WSR parameters in each cohort. Here, a detailed analysis of microcirculatory oxygen extraction in forearm skeletal muscle during cuff occlusion and subsequent rate of increase in oxygen saturation upon cuff release was undertaken to determine their associations with hyperemic WSR parameters. As part of the analysis, a microcirculatory oxygen saturation parameter that was associated with hyperemic WSR parameters was divided into subgroups based on the above/below median values. These subgroups were then compared to see whether there was a difference in BA hyperemic WSR parameters between subgroups. Finally, we determined associations between microcirculatory oxygen saturation parameters and the magnitude of BAFMD in each cohort. Our hypothesis was that microcirculatory oxygen saturation parameters during ischemia and upon reperfusion would be associated with brachial artery hyperemic WSR parameters in the young cohort, but not in the older cohort.

## METHODS

Healthy young (*n* = 17, 6 females, aged ≥20 and <40 yr) and older adults (*n* = 32, 16 females, aged ≥60 yr old) participated in this study. Exclusion criteria were hypertension, type 2 diabetes, dyslipidemia, or overt cardiovascular disease, and taking medications for cardiovascular risk modification. United Kingdom National Research Ethics Service South West Committee (10/H0203/29) approved all study procedures, and all the participants provided written informed consent.

Participants arrived in a temperature-controlled laboratory (NIHR Exeter Clinical Research Facility, Exeter, UK) after an overnight fast. Upon arrival, they had blood samples drawn for biochemical analysis, had a standardized breakfast, and were acclimatized for 20 min supine on an examination bed before initiation of the study protocol.

### Brachial Artery Flow-Mediated Dilation

The detail of our BAFMD assessment, as per established guidelines (8), has been previously described (4, 9). Participants lay supine on the examination bed with the right arm fixed in position and immobilized using a positioning pillow on a sturdy metal table. A small blood pressure cuff was placed around the proximal forearm. B-mode ultrasound images and multigate Doppler blood velocity data from BA were obtained using the Ultrasound Advanced Open Platform (Microelectronics Systems Design Laboratory, University of Florence, Italy) with a high-frequency linear array transducer (LA523; Esaote SpA, Florence, Italy) as described previously (4, 9). Once the participant had acclimatized, the transducer was carefully clamped over BA using a custom-designed transducer holder. Following a steady baseline of a minimum of 15 min for all macro- and microvascular parameters, the baseline BA image and blood velocity were obtained for 60 s before the forearm cuff was rapidly inflated to 250 mmHg to occlude forearm blood flow for 5 min using an arterial inflow system (AI6; Hokanson, Bellevue, WA). The recording of BA images and blood velocity was resumed 30 s before cuff deflation. The cuff was then rapidly deflated at 5 min to induce reactive hyperemia while recording was continued until 5 min after deflation.

### Near-Infrared Spectroscopy

Simultaneously with BAFMD, forearm microcirculatory oxygen saturation of forearm skeletal muscle was assessed using NIRS (Hamamatsu NIRO 200, Iwata, Japan) (10). A near-infrared probe was placed over the flexor digitorum profundus of the right arm below the blood pressure cuff being placed to prevent movement artifacts. The light source and detector were spaced 4 cm apart, giving a tissue sample volume to a depth of ∼2 cm. The sampled volume of tissue is considered to be to a depth of approximately half the probe spacing, from which a measure of the mean blood oxygen saturation can be derived as follows: mean blood oxygen saturation = [Hbo_2_] × 100/([Hbo_2_] + [Hb]) where [Hb] and [Hbo_2_] are relative changes in concentrations of deoxyhemoglobin and oxyhemoglobin, respectively. The sum of the [Hbo_2_] and [Hb] components provides a measure of blood volume change over the course of the study. Recording of NIRS data was started once the ultrasound probe had been clamped securely and continued thereafter until the end of BAFMD assessment.

### Data Analysis

#### Brachial artery parameters.

As described previously (4, 9), detailed WSR and diameter parameters were extracted using a custom-designed signal elaboration system (11) and are described in Supplemental Fig. S1 (all Supplemental Figures and Tables can be accessed at https://doi.org/10.6084/m9.figshare.25866904). For this study, the following WSR parameters relevant to reactive hyperemia were analyzed: WSR at peak hyperemia (WSR PK), area under the WSR curve until time to peak dilation (WSR_aucttp_), area under the WSR curve until time to return to baseline value (WSR_auc_) and first slope of WSR increase (WSR SL1). In addition, baseline diameter, absolute diameter increase from baseline (ΔDiam), and percent diameter increase from baseline (%ΔDiam) were analyzed.

#### Microcirculatory oxygen saturation parameters.

Following parameters were identified as relevant to microvascular function during ischemia-reperfusion based on literature and our previous publication (2, 12): baseline oxygen saturation (So_2base_), oxygen extraction during ischemia (OE), minimum oxygen saturation during ischemia (So_2min_), area enclosed by oxygen saturation signal during ischemia and baseline level (So_2deficit_), time delay before oxygen saturation increase during reperfusion (So_2td_), time to peak oxygen saturation during reperfusion (So_2tp_), peak oxygen saturation during reperfusion (So_2peak_), and oxygen saturation increase slope during early reperfusion (So_2slope_). The description of these microvascular parameters is listed in Supplemental Table S1, and a schematic example of these parameters is presented in Supplemental Fig. S1.

Data are presented as means ± SD and medians [interquartile ranges]. A χ^2^ test was used to examine differences in categorical variables. An independent samples *t* test and Mann–Whitney test were used to compare continuous variables. Pearson correlation coefficient and Spearman’s rank correlation coefficient were performed separately for young and older cohorts to determine associations between macro- and microvascular parameters. We did not perform the allometric scaling for BAFMD as baseline BA diameter was similar between cohorts with comparable sex proportions (Table 1). Statistical analysis was performed using SPSS (v.28, IBM, Armonk, NY), and *P* < 0.05 was considered statistically significant.

**Table 1.** Comparison of selected characteristics and macro- and microvascular parameters analyzed in this study between cohorts

	Young	Older	*P* Value
*n*	17	32	
Characteristics			
Age, yr	28.7 ± 6.7	64.9 ± 3.7	<0.001
Female, *n*	6	16	0.325
BMI, kg/m^2^	23.6 ± 2.3	24.6 ± 2.5	0.090
Cholesterol, mmol/L			
Total	4.5 ± 0.8	6.0 ± 1.4	<0.001
HDL	1.8 ± 0.4	1.7 ± 0.5	0.755
HbA1c, mmol/mol	36 ± 3	40 ± 4	<0.001
Blood pressure, mmHg			
Systolic	116 ± 9	124 ± 13	0.011
Diastolic	68 ± 7	73 ± 7	0.028
Heart rate, beats/min	62 ± 9	60 ± 8	0.280
Macrovascular			
WSR PK, s^−1^	636 ± 132	465 ± 108	<0.001
WSR_aucttp_, AU	14,917 ± 5,881	11,198 ± 4,482	0.017
WSR_auc_, AU	18,603 ± 7,000	14,286 ± 4,877	0.015
WSR SL1, s^−2^	90.9 ± 14.5	68.2 ± 18.2	<0.001
Diam_base_, mm	3.41 ± 0.52	3.60 ± 0.70	0.304
ΔDiam, mm	0.24 ± 0.09	0.16 ± 0.10	0.008
%ΔDiam, %	7.2 ± 2.6	4.9 ± 3.2	0.013
Microvascular			
So_2base_, %	73.7 [63.5–79.9]	72.1 [67.2–74.7]	0.450
OE, %s^−1^*	0.15 [0.12–0.17]	0.09 [0.05–0.12]	0.003
So_2min_, %	53.5 [41.3–60.6]	56.4 [50.1–63.1]	0.147
So_2deficit_, %s	3,810 ± 1,420	2,723 ± 1,240	0.008
So_2td_, s	2.4 [1.8–3.1]	2.9 [1.7–3.9]	0.207
So_2tp_, s	25.8 ± 6.3	26.6 ± 6.2	0.660
So_2peak_, %	86.7 [82.0–88.2]	83.7 [78.9–86.8]	0.095
So_2slope_, %s^−1^	2.5 ± 0.7	1.7 ± 0.7	<0.001

Values are means ± SD and medians [interquartile ranges]; *n*, number of participants in group. *n = 16 for both cohorts. BMI, body mass index; HDL, high-density lipoprotein; HbA1c, hemoglobin A1c; WSR PK, WSR at peak hyperemia; WSR_aucttp_, area under the WSR curve until time to peak dilation; WSR_auc_, area under the WSR curve until time to return to baseline value; WSR SL1, first slope of WSR increase; Diam_base_, baseline diameter; ΔDiam, absolute diameter increase from baseline; %ΔDiam, percent diameter increase from baseline. So_2base_, baseline oxygen saturation; OE, oxygen extraction during ischemia; So_2min_, minimum oxygen saturation during ischemia; So_2deficit_, area enclosed by oxygen saturation signal during ischemia and baseline level; So_2td_, time delay before oxygen saturation increase during reperfusion; So_2tp_, time to peak oxygen saturation during reperfusion; So_2peak_, peak oxygen saturation during reperfusion; So_2slope_, oxygen saturation increase slope during early reperfusion.

## RESULTS

Selected characteristics of the study participants are shown in Table 1. Those data from the older cohort have been previously reported (4). When compared with the young cohort, the older cohort had higher total cholesterol, higher hemoglobin A1c concentration, and higher systolic and diastolic blood pressure (all *P* < 0.05).

Table 1 shows macrovascular parameters analyzed in this study between cohorts. All hyperemic WSR parameters were smaller in the older cohort than the young cohort (all *P* < 0.05). In addition, ΔDiam and %ΔDiam were significantly lower in the older cohort than the young cohort (both *P* < 0.05). Baseline BA diameter was not statistically different between cohorts (*P* = 0.304). Microvascular parameters analyzed between cohorts are also shown in Table 1. During ischemia, OE was lower in the older cohort than the young cohort (*P* = 0.003). Likewise, So_2deficit_ was smaller in the older cohort than the young cohort (*P* = 0.008). Upon reperfusion, So_2slope_ was lower in the older cohort than the young cohort (*P* < 0.001). So_2peak_ tended to be lower in the older cohort (*P* = 0.095). Supplemental Table S2 reports an exploratory analysis comparing macro- and microvascular parameters analyzed in this study stratified by sex.

**Figure 1.**
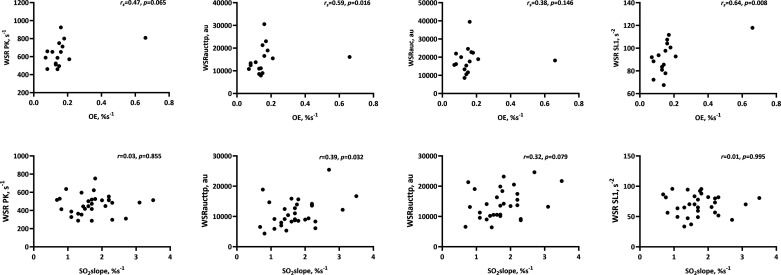
Associations between oxygen extraction during ischemia (in the young cohort, *n* = 16), oxygen saturation increases slope during early reperfusion (in the older cohort, *n* = 32), and brachial artery hyperemic WSR parameters. OE, oxygen extraction during ischemia; *r_s_*, Spearman’s rank correlation coefficient; So_2slope_, oxygen saturation increase slope during early reperfusion; WSR PK, WSR at peak hyperemia; WSR SL1, first slope of WSR increase; WSR_auc_, area under the WSR curve until time to return to baseline value; WSR_aucttp_, area under the WSR curve until time to peak dilation.

Figure 1 and Table 2 show associations between microcirculatory oxygen saturation parameters and BA hyperemic WSR parameters in the young cohort. From this analysis onward, microcirculatory parameters are limited to those that were significantly (i.e., *P* < 0.05) or tended to be (i.e., *P* < 0.10) different between cohorts (Table 1). OE was associated with WSR_aucttp_ and WSR SL1 (both *P* < 0.05, Fig. 1). OE also tended to be associated with WSR PK (*P* = 0.065, Table 2). So_2peak_ showed a tendency for association with WSR_aucttp_ (*P* = 0.073, Table 2). No other association was observed in this cohort. Figure 1 and Table 2 also show associations between microcirculatory oxygen saturation parameters and BA hyperemic WSR parameters in the older cohort. So_2slope_ was associated with WSR_aucttp_ (*P* = 0.032, Fig. 1) and tended to be associated with WSR_auc_ (*P* = 0.079, Table 2). Other microcirculatory oxygen saturation parameters did not show an association with BA hyperemic WSR parameters in the older cohort.

**Table 2.** Associations between microcirculatory oxygen saturation parameters and brachial artery hyperemic WSR parameters in young and older cohorts

		WSR PK		WSR_aucttp_		WSR_auc_		WSR SL1	
	*n*	*r*	*P* value	*r*	*P* value	*r*	*P* value	*r*	*P* value
Young	17								
So_2deficit_		0.01	0.957	0.20	0.433	0.04	0.876	0.24	0.352
So_2peak_*		0.23	0.374	0.45	0.073	0.23	0.384	0.40	0.110
So_2slope_		0.28	0.280	0.36	0.162	0.32	0.212	0.14	0.603
Older	32								
OE	16	−0.27	0.321	−0.23	0.396	−0.19	0.475	−0.25	0.351
So_2deficit_		−0.20	0.278	0.03	0.861	−0.15	0.402	−0.17	0.354
So_2peak_*		−0.07	0.692	0.20	0.275	0.19	0.319	−0.03	0.864

* Spearman’s rank correlation coefficient (*r*_s_) values are presented for So_2peak_ in both cohorts; *n*, number of participants in group. WSR PK, WSR at peak hyperemia; WSR_aucttp_, area under the WSR curve until time to peak dilation; WSR_auc_, area under the WSR curve until time to return to baseline value; WSR SL1, first slope of WSR increase; OE, oxygen extraction during ischemia; So_2deficit_, area enclosed by oxygen saturation signal during ischemia and baseline level; So_2peak_, peak oxygen saturation during reperfusion; So_2slope_, oxygen saturation increase slope during early reperfusion.

Because our sample size was relatively small, we performed a sensitivity analysis with the microvascular parameters that were associated with BA hyperemic WSR parameters in the correlation analysis in each cohort. BA hyperemic WSR parameters were then compared between above/below median values of OE in the young cohort and between above/below median values of So_2slope_ in the older cohort (Supplemental Fig. S2). In the young cohort, all BA hyperemic WSR parameters were different between those with OE above median and those with OE below median. Specifically, WSR PK (731 [592–807] vs. 556 [475–637] s^−1^), WSR_aucttp_ (17,756 [15,630–22,588] vs. 11,036 [9,135–13,126] arbitrary units, AU), and WSR_auc_ (20,663 [17,800–24,106] vs. 15,562 [11,239–19,063] AU) were all greater in those with OE above median than those below median (all *P* < 0.05). Similarly, WSR SL1 was higher in those with OE above median compared with those below median (102.3 [93.9–110.6] vs. 84.5 [74.4–91.1] s^−2^, *P* = 0.009). In the older cohort, WSR_aucttp_ (12,474 [8,913–14,028] AU, *P* = 0.081) and WSR_auc_ (14,859 [12,568–20,057] AU, *P* = 0.053) tended to be greater in those with So_2slope_ above median compared with those below median (Supplemental Fig. S2). WSR PK and WSR SL1 were both similar between subgroups in the older cohort.

Associations between microcirculatory oxygen saturation parameters and the magnitude of BA vasodilation in each cohort are presented in Table 3. In the young cohort, So_2peak_ was associated with both ΔDiam and %ΔDiam (both *P* < 0.05). In contrast, no association was observed between microcirculatory oxygen saturation parameters and the magnitude of BA vasodilation in the older cohort.

**Table 3.** Associations between microcirculatory oxygen saturation parameters and absolute and percent brachial artery vasodilation stratified by cohorts

		ΔDiam		%ΔDiam	
	*n*	*r*	*P* value	*r*	*P* value
Young	17				
OE*	16	0.17	0.541	0.22	0.413
So_2deficit_		0.35	0.176	0.21	0.418
So_2peak_*		0.65	0.005	0.71	0.001
So_2slope_		0.06	0.809	−0.03	0.911
Older	32				
OE	16	−0.04	0.872	−0.14	0.598
So_2deficit_		−0.07	0.722	−0.08	0.668
So_2peak_*		0.27	0.139	0.21	0.255
So_2slope_		0.08	0.665	0.04	0.848

* Spearman’s rank correlation coefficient (*r*_s_) values are presented for OE in young cohort and So_2peak_ in both cohorts; *n*, number of participants in group. ΔDiam, absolute diameter increase from baseline; %ΔDiam, percent diameter increase from baseline; OE, oxygen extraction during ischemia; So_2deficit_, area enclosed by oxygen saturation signal during ischemia and baseline level; So_2peak_, peak oxygen saturation during reperfusion; So_2slope_, oxygen saturation increase slope during early reperfusion.

## DISCUSSION

The salient findings of this study are as follows. Oxygen extraction (i.e., OE) and oxygen consumption during ischemia (i.e., So_2deficit_) parameters were significantly lower in the older cohort than the young cohort. Similarly, oxygen resaturation kinetics upon reperfusion (i.e., So_2slope_) was significantly lower in the older cohort compared with the young. Moreover, OE was significantly associated with BA hyperemic WSR parameters (WSR_aucttp_ and WSR SL1) in the young cohort. In the older cohort, So_2slope_ was significantly associated with WSR_aucttp_. When OE and So_2slope_ were divided into their above/below median values, all BA hyperemic WSR parameters were significantly greater in those with OE above the median than those with OE below the median in the young cohort, whereas in the older cohort, hyperemic WSR parameters were not statistically different between those with So_2slope_ above the median and those with So_2slope_ below the median. Finally, we observed an association between So_2peak_ and BAFMD in the young cohort, but it did not reflect the stimulus-response relationship between microvascular response to ischemia and hyperemic WSR in the young cohort. Overall, these findings reveal, for the first time to our knowledge, interactions between micro (stimulus)- and macrocirculation (response) to produce BAFMD in humans. Furthermore, the findings suggest that a reduced BAFMD observed in older adults may be a result of multiple alterations occurring not solely at macrocirculation but both at macro- and microcirculation.

Vascular endothelium plays a pivotal role in human cardiovascular aging. Impaired endothelial function with aging is apparent even without concomitant cardiovascular risk factors (13). BAFMD quantifies the magnitude of BA diameter change in response to reactive hyperemia (1). Although a BA diameter change can be precisely measured, BAFMD has continuously struggled to precisely estimate WSR near the arterial wall throughout its procedure (9), leaving ambiguity in interpreting a reduced BAFMD observed with aging: it is unclear if this is due to an altered WSR or due to an altered vasodilatory response to WSR stimulus (9, 14). Technical advancement in the field of ultrasound was able to overcome this shortcoming (9), and we have previously reported that healthy older adults not only exhibit a diminished hyperemic WSR but also exhibit a dissociation between WSR and BA vasodilatory response, which may reflect a loss of precision in the reactive hyperemia stimulus-response relationship (4). These observations then raise a question as to what influences BA hyperemic WSR response in healthy young and older adults.

With a brief period of ischemia, forearm microvessels experience an incremental oxygen supply-demand mismatch, causing a fall in blood oxygen saturation signal measured by NIRS as the ischemic stimulus continues. Because of a build-up of metabolic byproducts and adenosine triphosphate released by erythrocytes (15) in microvessels under ischemia, these vessels respond by vasodilation (16). The extent of vasodilation and the subsequent reperfusion influences blood flow in BA, and hence its WSR (3). Indeed, our older adults had significantly lower OE and So_2deficit_ during ischemia, and lower So_2slope_ upon reperfusion than our young counterparts. A diminished microvascular response to ischemia and an altered microvascular reperfusion could reduce BA hyperemic WSR during reperfusion. The mechanisms downstream of hyperemic WSR including structural alterations in the microvessels (17), age-associated microvascular endothelial dysfunction (18), diminished microvascular glycocalyx and mechanotransduction (19), and an altered microvascular autoregulatory response to reperfusion (2) can influence the microvessel’s ability to respond to ischemia reperfusion. Furthermore, an age-associated decline in skeletal muscle oxidative function may contribute to diminished oxygen extraction during ischemia, which is likely attributed to mitochondrial dysfunction and/or diffusive oxygen conductance limitation (12). These mechanisms, likely in combination, could reduce the magnitude of vasodilation during ischemia and the rate of reperfusion in microvessels, resulting in a diminished hyperemic WSR observed in our older cohort.

Further analysis revealed a positive association between OE and BA hyperemic WSR parameters in the young cohort. The finding became more marked when OE data were stratified as above/below median value; all BA hyperemic WSR parameters were significantly greater in those with OE above the median compared with those below the median. These findings, together with the intact hyperemic WSR and BAFMD relationship (4), suggest that consecutive stimulus-response relationships (i.e., OE-hyperemic WSR and hyperemic WSR-BAFMD) may be intact to induce BAFMD in the young cohort. In contrast, So_2slope_ was positively associated with WSR_aucttp_ in the older cohort but, when the So_2slope_ data were stratified as above/below median value, there was no clear difference in WSR_aucttp_ or other hyperemic WSR parameters between So_2slope_ above/below median groups. This observation sheds new light on interpreting BAFMD in older adults; a reduced BAFMD is conventionally interpreted as BA endothelial dysfunction because of impairments in the endothelium occurring during the sequence of events from mechanotransduction of WSR stimulus to endothelium-derived nitric oxide synthesis, hyperpolarization, and the overall bioavailability of endothelial-derived relaxing and contracting factors to smooth muscle cells. However, we suggest that the reduced BAFMD observed in older adults may be a result of complex interactions attributed to *1*) a diminished microvascular dilatory response to ischemia and reduced reperfusion kinetics upon reperfusion, both of which contribute to an altered stimulus-response relationship to produce BA hyperemic WSR; *2*) a diminished BA hyperemic WSR response; and *3*) an altered stimulus-response relationship at the level of BA endothelium that could reduce the precision of BA vasodilatory response to the increased WSR stimulus. Therefore, the reduced BAFMD in older adults may not solely reflect BA endothelial dysfunction per se. Rather, it may well reflect a reduced ability of macro- and microvascular function to respond to ischemic stimulus and to transduce the stimulus to the next, resulting in the reduced BAFMD in this cohort.

Evidence of macro- and microvascular alterations to induce BAFMD in older adults has important implications for the accurate interpretation of BAFMD in general and also for our understanding of vascular aging. Specifically, given the altered microvascular dilatory response to ischemia and the inability to precisely transduce stimulus for producing hyperemic WSR, microvascular function in older adults may not properly reflect the magnitude of stimulus for BAFMD notwithstanding the dissociation of WSR-BAFMD relationship. The absence of association observed between microvascular oxygen saturation parameters and BAFMD in the older cohort supports this contention. Intriguingly, a positive association was observed between So_2peak_ and BAFMD in the young cohort, which seems to be plausible. However, the So_2peak_ value was not statistically different between cohorts and So_2peak_ was not associated with hyperemic WSR parameters in the young, which raises doubts about the legitimacy of the observed association. Therefore, caution should be exercised to interpret the association between microvascular function and BAFMD because of the complex interaction between consecutive stimulus-response relationships in the process of BAFMD.

There are some limitations in this study. The number of participants was relatively small, and because we studied only healthy adults, our findings may not be generalizable to other populations. In addition, we did not apply multiple testing corrections because the vascular parameters investigated are correlated and the Bonferroni correction is too conservative in this case. Finally, the data regarding physical activity and fitness levels were not available, which could have helped interpret our observations.

### Conclusion

This study demonstrates that microvascular response to ischemia and subsequent reperfusion is diminished in older adults. Furthermore, there appears to be a dissociation between microvascular response to ischemia and BA hyperemic WSR in older adults, which may further alter BAFMD. These findings suggest that a reduced BAFMD observed in older adults may be a result of multiple alterations occurring not solely at macrocirculation, but both at macro- and microcirculation. BAFMD may be an integrated indicator of systemic vascular function in healthy older adults reflecting macro- and microvascular cross talk.

## DATA AVAILABILITY

Data will be made available upon reasonable request.

## SUPPLEMENTAL DATA

10.6084/m9.figshare.25866904 Supplemental Tables S1–S2 and Supplemental Figs. S1–S2 can be accessed at https://doi.org/10.6084/m9.figshare.25866904.

## GRANTS

This study was supported by European Union’s Seventh Framework Programme FP7/2007–2013 for Innovative Medicine Initiative Grant IMI/115006 (the SUMMIT consortium) and partly by the National Institute of Health and Care Research (NIHR) Exeter Clinical Research Facility.

## DISCLAIMERS

The views expressed are those of the authors and not necessarily those of the National Health Service, NIHR, or the Department of Health.

## DISCLOSURES

No conflicts of interest, financial or otherwise, are declared by the authors.

## AUTHOR CONTRIBUTIONS

C.E.T., P.E.G., F.C., A.R., P.T., C.P., A.C.S., and K.A. conceived and designed research; C.E.T., P.E.G., F.C., and K.A. performed experiments; C.E.T., P.E.G., A.R., and K.A. analyzed data; C.E.T., P.E.G., A.R., C.P., A.C.S., and K.A. interpreted results of experiments; C.E.T. and K.A. prepared figures; K.A. drafted manuscript; C.E.T., P.E.G., F.C., A.R., P.T., C.P., A.C.S., and K.A. edited and revised manuscript; C.E.T., P.E.G., F.C., A.R., P.T., C.P., A.C.S., and K.A. approved final version of manuscript.
